# S8-5 INTERACT capacity building framework for sport for all among sports federations

**DOI:** 10.1093/eurpub/ckad133.042

**Published:** 2023-09-11

**Authors:** Game Mothibi

**Affiliations:** TAFISA

## Abstract

**Purpose:**

International Sports Organisations (ISOs) have increasing interest in investing on sport for all and have been called by policy-makers through different policies (e.g. WHO Global Action Plan for physical activity, UN Sustainable Development Goals) to leverage their health and physical activity potential towards the society. This presentation showcase a training (in-person, informal education workshop) that aims to empower the staff and volunteers of ISOs promote Sport for All and physical activity and act as multipliers to train their national federations and grassroot sport organisations.

**Project:**

The INTERACT Capacity-Building Framework which was designed by INTERACT Erasmus+ project partners (TAFISA and 8 International Sports Organisation) through different meetings and a co-construction approach, addresses key elements, identified as part of the INTERACT project, to support ISOs and continental/national sport federations in developing their own Sport for All and physical activity initiatives and engage with their members in the field. It provides exchange of knowledge and training to empower ISOs, their staff and volunteers in Sport for All and place physical activity promotion at the core of their activities. This will in turn increase participation in sport and physical activity.

By using different sensory channels (e.g. visual, acoustic, tactile), our approach satisfies the participant’s different learning types and primary ways of absorbing and processing information. Composed of three steps (advocacy, diagnostic, training), it alternates theory and practice as well as tension and relaxation, movement and rest. Participant-oriented educational work includes the conscious handling of diversity and diversity of people (e.g. in relation to sex/gender, nationality, ethnicity, religious belief, disability, sexual orientation etc.). A first pilot has been launched among 12 ISOs, and evaluation framework will be created this spring, based on a litterature review.

**Conclusion:**

The INTERACT Capacity-building programme is developed to become a full-fetched service that is offered in the coming years to any ISO willing to step up its promotion of Sport for All activity.

